# Skip-layer network with optimization method for domain adaptive detection

**DOI:** 10.1371/journal.pone.0263748

**Published:** 2022-03-17

**Authors:** Qian Xu, Ying Li, Gang Wang, Minghui Hou, Hao Zhang, Hongmin Cai

**Affiliations:** 1 College of Computer Science and Technology, Jilin University, Changchun, People’s Republic of China; 2 Key Laboratory of Symbolic Computation and Knowledge Engineering of Ministry of Education, Jilin University, Changchun, People’s Republic of China; 3 State Key Laboratory of Automotive Simulation and Control, Jilin University, Changchun, People’s Republic of China; 4 School of Computer Science and Engineering, South China University of Technology, Guangzhou, People’s Republic of China; University of Engineering & Technology, Taxila, PAKISTAN

## Abstract

In the field of object detection, domain adaptation is one of popular solution to align the distribution between the real scene (target domain) and the training scene (source domain) by adversarial training. However, only global features are applied to the Domain Adaptive Faster R-CNN (DA Faster R-CNN) method. The lack of local features reduces the performance of domain adaptation. Therefore, a novel method for domain adaptive detection called Skip-Layer Network with Optimization (SLNO) method is proposed in this paper. Three improvements are presented in SLNO. Firstly, different level convolutional features are fused by a multi-level features fusion component for domain classifier. Secondly, a multi-layer domain adaptation component is developed to align the image-level and the instance-level distributions simultaneously. Among this component, domain classifiers are used in both image-level and instance-level distributions through the skip layer. Thirdly, the cuckoo search (CS) optimization method is applied to search for the best coefficient of SLNO. As a result, the capability of domain alignment is strengthened. The Cityscapes, Foggy Cityscapes, SIM10K, KITTI data sets are applied to test our proposed novel approach. Consequently, excellent results are achieved by our proposed methods against state-of-the-art object detection methods. The results demonstrate our improvements are effective on domain adaptation detection.

## 1. Introduction

Recently, object detection is a research hotspot for scholars and industry in the computer vision field. Object detection aims at identifying and localizing all object instances of interest in an image. Especially, object detection for persons, cars, bicycles etc is widely used in the unmanned driving area [1]. Object detection technology is the fundamental task for environmental perception.

In the past ten years, the breakthrough for object detection has been achieved based on the Convolutional Neural Network (CNN) and the large-scale public data sets. The mainstream approaches can be divided into two categories by the number of stages named two-stage method and one-stage method. On the one hand, the most representative method of the two-stage method is the Region-based Convolutional Neural Network (R-CNN) series. Girshick et al. proposed a Region-based Convolutional Neural Network (R-CNN) for object detection in 2014 [2]. Great performance is got by combining the regions with CNN features. Furthermore, R-CNN’s detection capability and speed are strengthened by mapping the region proposals into the last layer feature maps of the CNN in Fast R-CNN [3]. Moreover, RPN is applied to replace the Selective Search (SS) [4] for generating the region proposals in Faster R-CNN [5]. On the other hand, the most representative methods of the one-stage method are SSD [6]and YOLO [7, 8] series. Both of them are directly classified and regressed by using the features extracted from CNN.

The state-of-the-art experimental results have been witnessed by excellent object detection methods in the benchmark datasets. Nevertheless, the images collected by sensors from the real world deviate from the training data in object appearance, backgrounds, illumination, image quality, etc. For example, the appearance of objects collected by the camera of automatic driving vehicles is variant at different places, seasons, times, and even weather. In other words, there is a domain shift between the training (source) and testing (target) images. Obviously, the generated model over the source domain can not be directly used to the unlabeled target domain. Moreover, as we know, annotation data can not be collected easily. In the real word, the proportion of labeled data is very small compared with unlabeled data. Therefore, the cost for labeling all the collected data is high.

The unsupervised domain adaptation method [9] is developed to adapt object detection models to the unlabeled target domains from the rich labeled source domains to solve the above problems. Usually, adversarial training is an important way to suit normal samples of neural network with disturbed samples. Therefore, the adversarial training method is introduced to minimize the divergence between the source and the target domains. In other words, the distribution of the source and target objects can be well aligned by finding the domain-invariant features of the objects.

DA Faster R-CNN [9] is a classical method among the mainstream domain adaptive detection methods. Both the image and instance distributions are aligned across domains based on adversarial training to solve the domain shift problems. Nowadays, DA Faster R-CNN has rapidly developed into a successful series. Saito et al. [10] and Zhu et al. [11] improved DA Faster R-CNN focused on image-level alignment forces to align non-transferable backgrounds. Furthermore, although instance-level domain classifiers can match region proposals in both domains, current methods, such as DA Faster RCNN [9], MA Model [11], PDA Model [12], DT Model [13] lack the ability to consider the lower-level feature maps with high resolution.

Three drawbacks of DA Faster R-CNN and related methods can be found based on the introduction for these methods. Firstly, features from low layers are not used for training the domain classifier. Therefore, the performance of the domain classifier is affected without context information. Secondly, the distributions between the source domain and target domain in image-level and instance-level are not effectively aligned. Thirdly, hyperparameters are defined by fine tuning experience. So the classification capability is affected by not choosing the best solution.

Three improvements are proposed in this work to solve the problem mentioned above. First, a multi-level features fusion method is introduced for domain adaptive object detection. The performance of the domain classifier is promoted based on rich sampling feature information from low-level layers in our proposed method. Second, a multi-layer domain adaptation method is designed to align the distributions between source and target domains in image-level and instance-level respectively. Furthermore, the loss functions are modified according to the novel network framework. Therefore, the ability to align the source and target domain shift is enhanced. Third, Cuckoo Search (CS) method [14] is applied to optimize a hyperparameter of loss function. Therefore, the capability of loss function is strengthened.

The effectiveness of our proposed method is evaluated on the Cityscapes [15], Foggy Cityscapes [16], SIM10K [17], KITTI [18] data sets. Experiments show that our proposed methods can significantly solve the domain shift problem. Furthermore, the performance of classification capability is enhanced by introducing the CS optimization method.

## 2. Related work

### 2.1. Domain adaptive object detection

Recently, domain adaptation has become highlighted thought for object detection to reduce domain discrepancy between the training and testing data. Because the cross-domain robustness of object detection could be improved by domain adaptation, thus Chen et al. [9] tackle the domain gap by designing image-level and instance-level components based on H-divergence theory. Saito et al. [10] proposed an object detector constructed using an unsupervised method to complete cross-domain tasks from label-rich to label-poor and verified the method on four datasets. Zhu et al. [11] reposition the focus of the adaptation process from global to local by mining the discriminative regions that are directly pertinent to object detection and aligning them across different domains. He et al. [12] proposes two feature alignment module with the scale reduction module (SRM) and weighted gradient reversal layer (WGRL) for domain adaptive object detector. Hsu et al. [13] propose to bridge the domain gap with an intermediate domain and then progressively solve more manageable adaptation subtasks. Inoue et al. [19] proposed a new task framework for cross-domain supervised object detection, which can detect everyday objects in various image domains without instance-level annotations and significantly improves the average accuracy on the three image domain datasets. What`s more, Kim et al. [20] introduced a new unsupervised domain adaptation method for object detection. The goal of this work is to alleviate the imperfect translation problem of pixel-level adaptation and the source-biased discrimination problem of feature-level adaptation at the same time. And the mean average precision of this method on various datasets is better than the SOTA methods. More recently, Cai et al. [21] proposed the MTOR model in response to the high generalization error of the synthetic image model on the real image after the domain transfer and achieved a new record of a single model in a wide range of experiments. Xie et al. [22] propose a multi-level domain adaptive model to align the distributions of local-level features and global-level features simultaneously. Xu et al. [23] propose a categorical regularization framework for alleviating overlooking problem across domains and get prominent results.

### 2.2. Domain adaptive faster R-CNN

Our work is developed based on DA Faster R-CNN [9], which contains three components: Image-level Adaptation, Instance-level Adaptation and Consistency Regularization.

#### 2.2.1 Image-level adaptation

A domain classifier is trained to predict the domain label for each image patch. In this way, the domain shift caused by the image-level difference such as image style, scale, illumination, etc. is reduced.

#### 2.2.2 Instance-level adaptation

A domain classifier is trained for the feature vectors to align the instance-level distribution, such as object appearance, size, viewpoint etc.

#### 2.2.3 Consistency regularization

To learn the cross-domain robustness of bounding box predictor, consistency between the domain classifier on different levels needs to be enforced.

### 2.3. Cuckoo search optimization method

Cuckoo Search (CS) [14] is a heuristic algorithm proposed by Yang and Deb. The algorithm simulates the parasitic brooding process of the cuckoo and introduces the Levy flight [24] mechanism to update the nest position, which can quickly and effectively solve the optimization problem.

In order to simulate the breeding mode of the cuckoo, three assumptions are made to introduce the cuckoo algorithm: 1) each cuckoo can only lay one egg at a time and randomly select a nest to hatch; 2) The nest with the best eggs are kept to the next generation; 3) The number of nests is fixed, and the probability of host finding cuckoo eggs is *P*_*a*_. Specifically, the process of the algorithm is described as follows. First, a certain number of nests is determined, and cuckoos lay eggs in them. The solution space of the objective function includes eggs and the optimal nest, which is determined by the quality of the stored eggs. Secondly, the host abandons the egg or abandons the nest to build a new one after some eggs are found by the host.

**Fig 1:**
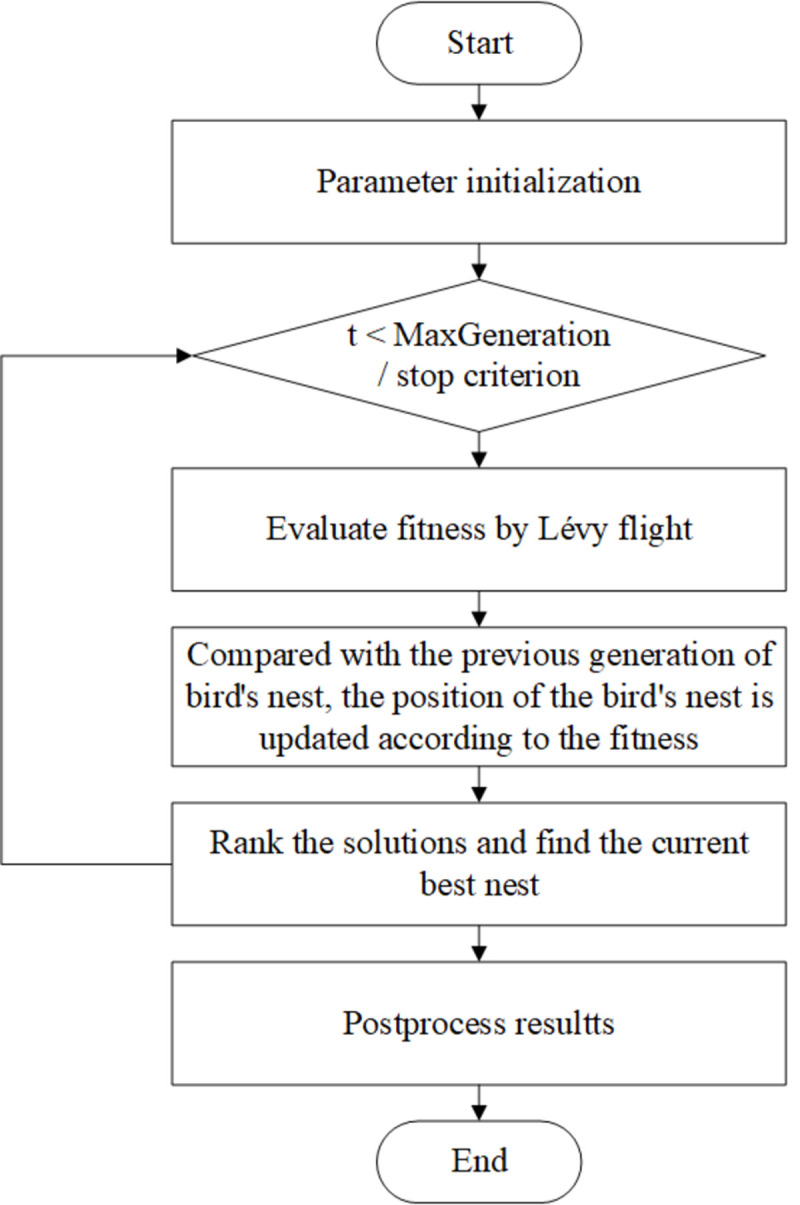
The flowchart of the cuckoo search method.

Finally, the best eggs can be obtained through multiple iterations of the above process by evaluating all the nests. In other words, the optimal solution for the objective function can be found. The flowchart is as shown in Fig 1.

## 3. Approach

### 3.1. Overview

In section 3.2, the framework of our proposed novel skip-layer method is introduced. In section 3.3, a multi-level features fusion method is illustrated. In section 3.4, the multi-layer domain adaptation is applied to align the distributions between domains in both image-level and instance-level. In section 3.5, loss functions are modified based on the skip-layer improvements. Finally, in section 3.6, the parameters of the loss function are optimized by the CS method.

### 3.2. Proposed skip-layer network

**Fig 2:**
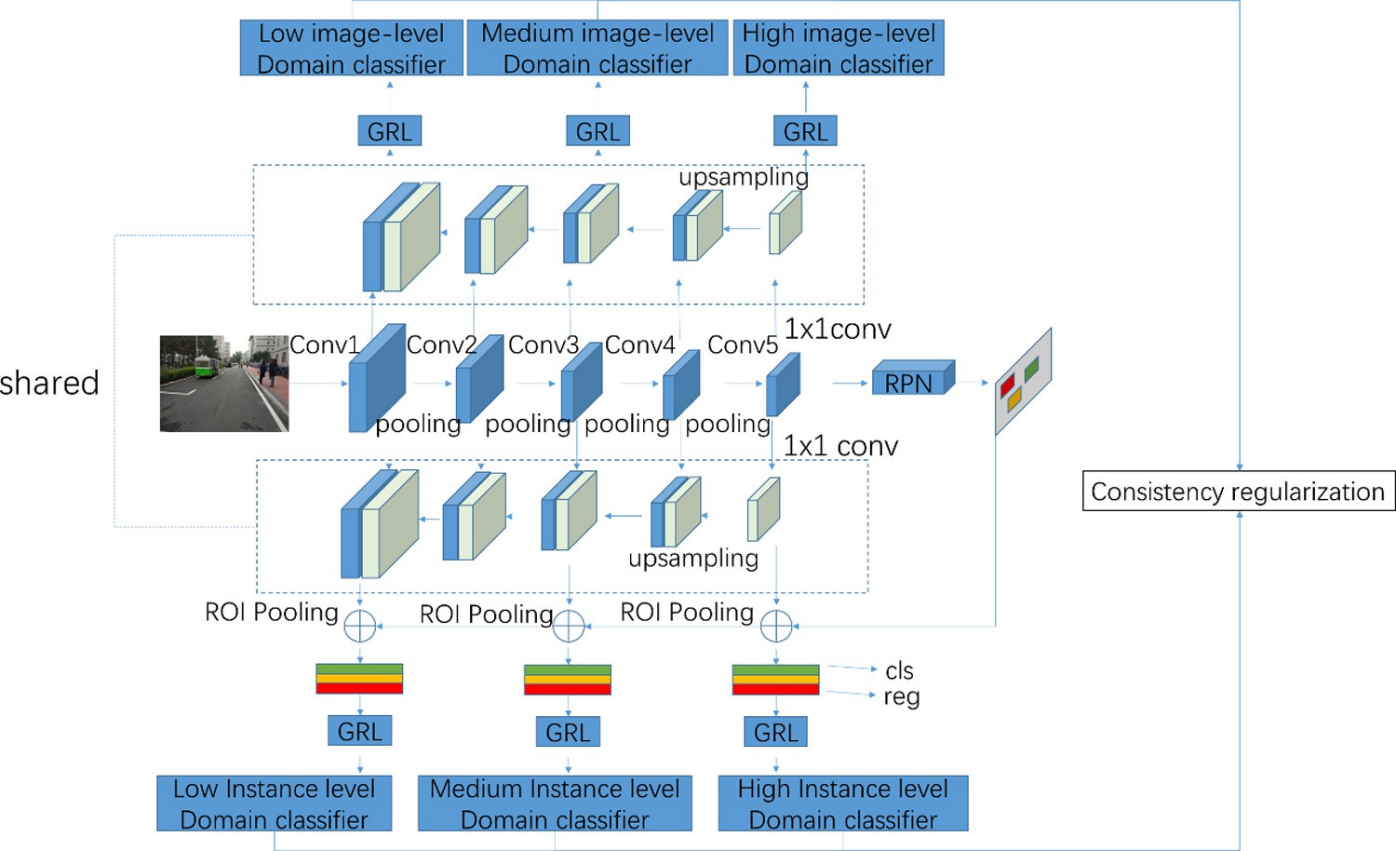
The framework of skip-layer network.

The top-level feature maps of the convolutional layer are applied to the domain classifier in DA Faster R-CNN [9]. However, the resolution for top-level feature maps is low. In addition, the lower-level feature maps with high resolution are not considered. Because the lower and higher feature maps are not contained in the domain classifiers, the adaptive model’s generalization ability is weakened. Furthermore, the distribution bias between the source and target domains is not reduced effectively by domain classifiers with only top-level feature maps. In order to solve the problems mentioned above, Skip-Layer Network is developed to enrich the information of domain classifiers in this paper. The framework of Skip-Layer Network is illustrated in Fig 2.

Two improvements are designed to promote the capability of domain classifiers. Firstly, a multi-level features fusion method is introduced to fuse the different convolution layer feature maps for domain classifiers. Secondly, a multi-layer domain adaptation method is developed to reduce the distribution bias between the source and target domains. The details of two improvements are described as follows.

### 3.3. Multi-level features fusion

**Fig 3:**
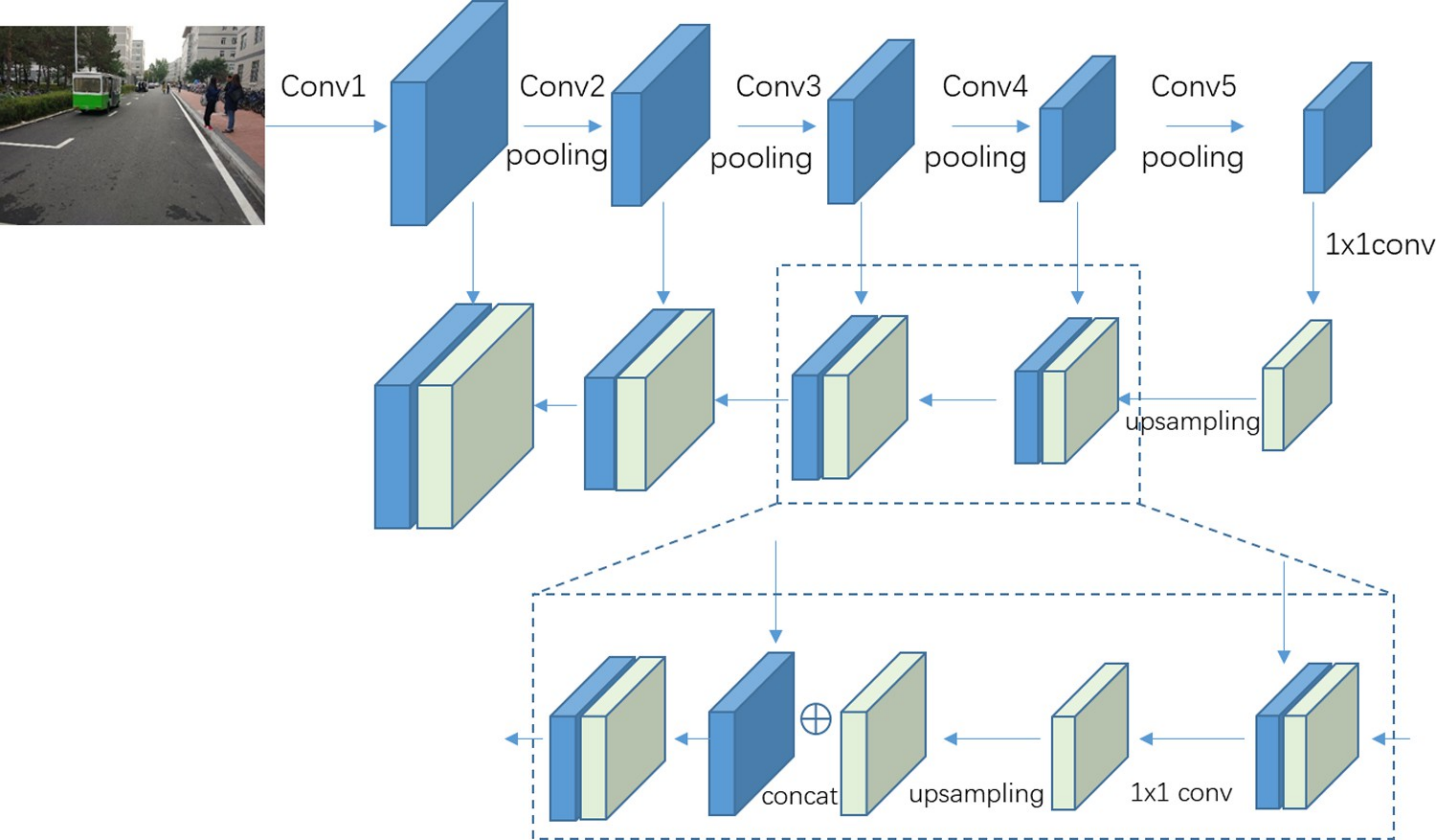
The multi-level feature fusion method.

In the classical forward convolutional propagation process, the overfitting problem is solved by introducing the pooling method. Kernel size *N* in the pooling method is applied to sample the convolutional feature maps. As a result, rich semantic information is contained in the higher-level feature maps. Additionally, rich pixel information is possessed in the lower-level feature maps. Thereupon, each level of convolutional feature maps has its limitations. Inspired by the feature pyramid networks [25], we believe the fused features from different convolutional layers contains more informations than single layer. More informations will be benefit to improve the performance of the domain classification. Therefore, the multi-level features fusion method is proposed to solve the abovementioned problems. For example, as shown in Fig 3, the feature maps from top to bottom is the feature maps from conv5, conv4, conv3, conv2 and conv1 sequentially. Because the top-level feature maps possess rich semantic information, thus top-level feature maps are applied to enhance each level of feature maps by the upsampling method. Two methods are introduced in this section to complete the fusion. Firstly, the channel consistency method is used to process the channel differences problem between the connected convolutional layers.

The higher-level convolutional layers have more channels than the lower-level convolutional layers. Therefore, the upsampling results cannot be concatenated to the current level feature maps directly. In our proposed method, 1x1 convolution layers are introduced to adjust the number of convolutional layer channels. The 1x1 convolution layer could change the channels feature maps to make the number of channels between connected convolutional layers the same.

Secondly the lower-level feature maps are sampled in the forward propagation by pooling layer, then the size of higher-level feature maps is smaller than the size of lower-level feature maps. However, the size of expansion results must be the same as the size of feature maps. Then, the concatenated method can be used to merge the two types of feature maps. In this situation, upsampling is introduced to expand the size of higher-level feature maps to fit the lower-level feature maps. The kernel size of the upsampling result is the same as the kernel size of the forward propagation pooling layers. Thereafter, the feature maps are unified to the same size between the higher and lower layers. In other words, the connected convolutional feature maps can be fused.

Finally, the information for each level of convolutional feature maps are strengthened by the multi-level features fusion component. As a result, the ability of domain adaptive classifier is enhanced.

### 3.4. Multi-layer domain adaptation

The domain classifier does not effectively solve the distribution deviation between the source domain and the target domain in DA Faster R-CNN. The reason is that the context convolutional layer information is not included in the domain classifier. Inspired by SharpMask [26], high-level information and low-level pixel data are both important for object detection. Particularly, rich spatial information is captured by lower layers in convolutional net. Meanwhile, object-level knowledge is extracted by upper layers, which factors such as pose and appearance are invariant. Then taking advantages of different levels information could also enhance the ability of domain classifier. This paper proposes a multi-layer domain adaptive method by applying the domain adaptive classifier through skip layers to solve this problem.

As shown in Fig 2, the multi-layer domain adaptation is used in both image-level and instance-level at the same time. Besides, the multi-level features fusion results are shared by the image-level and instance-level domain classifiers. In other words, the fused feature maps need to be calculated only one time from top to bottom. Nevertheless, the multi-layer domain adaptation is applied independently through skip layers. More details are described as follows.

**Fig 4:**
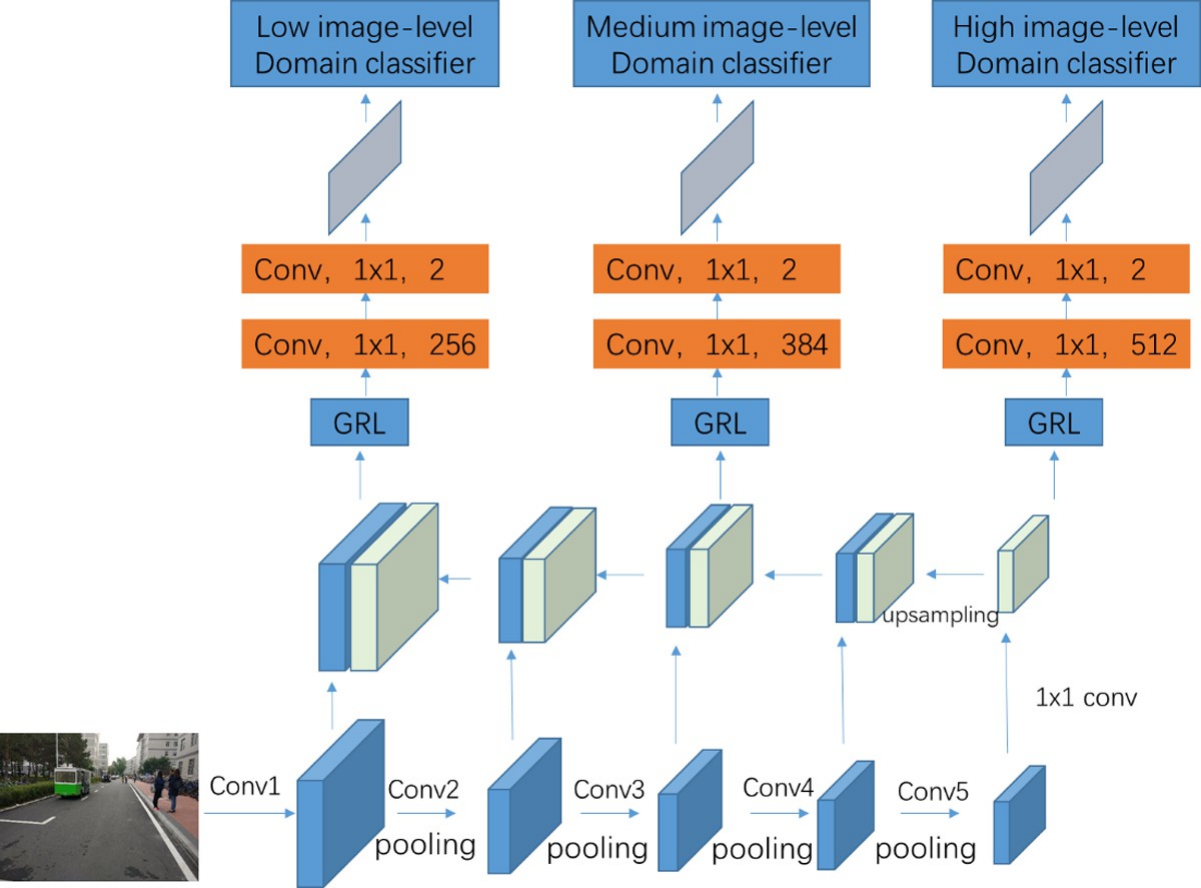
The detail architectures of the multiple image-level adaptation.

As shown in Fig 4, multiple image-level domain classifiers are built to reduce the domain distance in the corresponding image-level representation. Firstly, the reverse gradient layer (GRL) [27] is used to align the source domain and the target domain for minimizing the domain distance. Then, domain adaptive in DA Faster R-CNN is implemented by adversarial training. In other words, the loss result is minimized for the domain adaptive classifier while the loss result is maximized for the base detection network. In our proposed method, two fully convolution layers with kernel size 1x1 are added after GRL layer. Because the lower-level feature maps with high resolution contain richer context information, thus the number of output channels for the lower-level convolutional layer could less than that for the higher-level convolutional layer. In this way, the number of channels for the first convolution layer is decreased from 512 to 256. Additionally, the training time could be decreased for less calculation as well. Two channels are defined for the second convolution layer. At last, the loss could be calculated after a softmax layer and the gradients by backpropagate method.

Our proposed method has three advantages. First, the features of the adjacent layers are similar to each other. So the redundant problem is produced when aligning the domain distribution layer by layer. The skip architecture could avoid the problem effectively. Second, the skip architecture is a compromise of performance and training time. The parameters of model should be trained from random initialization. Therefore, the training time is long when using each convolutional layer. However, the training time can be reduced by applying the skip layer framework. Third, the adversarial training strategy can be implemented with less adaptive domain classifiers. And the convergence of model is easier in the training phase.

**Fig 5:**
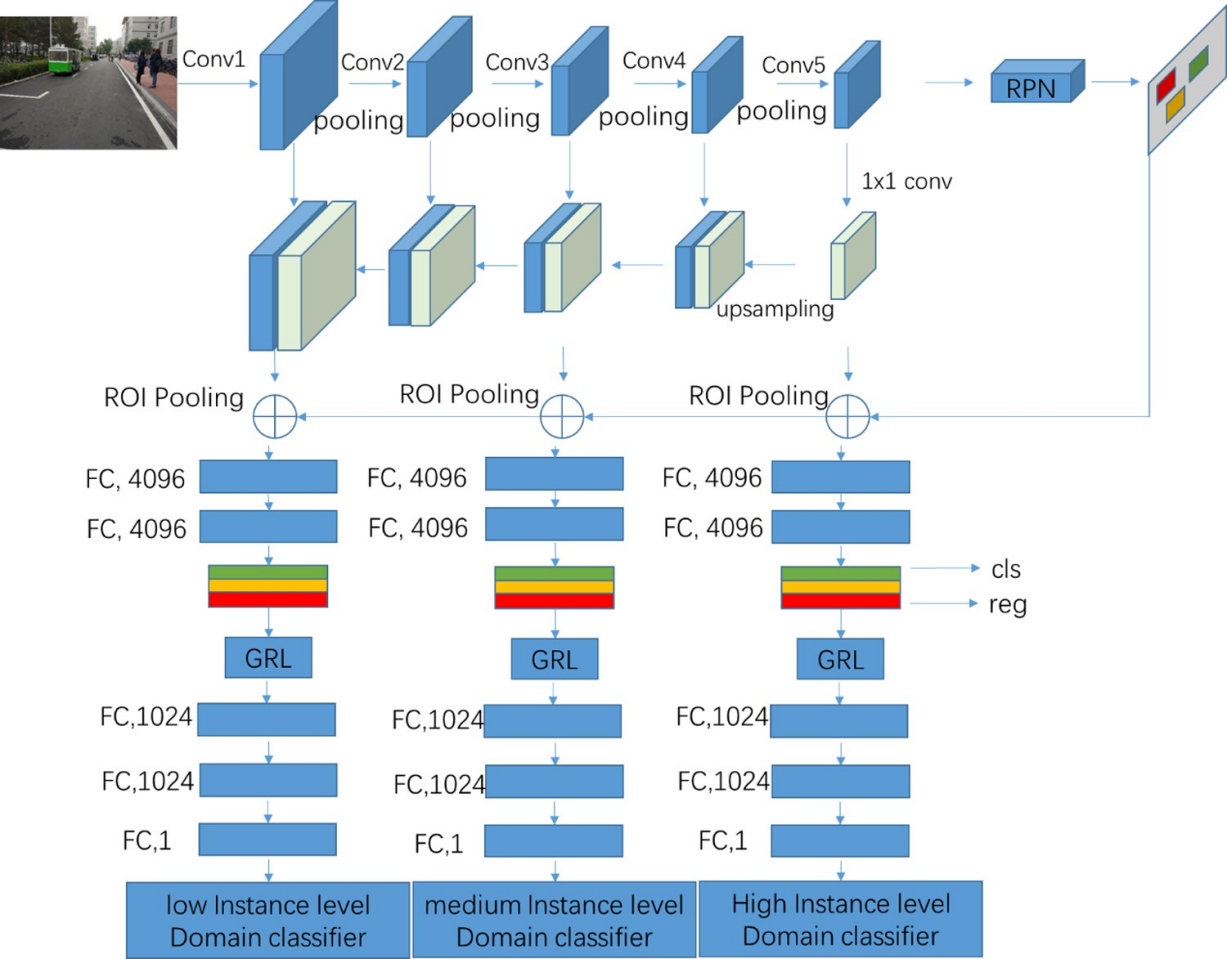
The detail architecture of instance level adaptation.

Multiple instance-level adaptation is developed to enhance the ability of the instance feature alignments. As shown in Fig 5, the region proposals are mapped from ground truth to the feature maps and from RPN to the feature maps for the source and target domains. Thereafter, two fully connection layers with 4096 output nodes are added to extract the instance-level information from the ROI-based feature vectors. In other words, the extracted features are linked to the instance level domain classifier component by GRL. Additionally, three fully connection layers with 1024, 1024 and1 output nodes are added sequentially. All the fully connection layers are activated by ReLU. In addition, these layers are generalized by Dropout with 0.5 ratio. Identically, all the instance-level domain classifiers’ architecture through low-level layers to high-level layers is the same. The size of feature maps at different layers is unified after ROI pooling. The weights of all the instance-level domain adaptive classifiers through low to high are optimized, respectively.

Obviously, the same instances are classified with different level features through the skip layers. The low-level feature maps with rich context information are applied to align domain distribution in our proposed methods. Therefore, the aligning domain distribution is better than the DA Faster R-CNN.

### 3.5. Modified loss functions

According to the improvements mentioned above, the loss functions of the original DA Faster R-CNN need to be modified to fit the skip-layer network. The multiple image-level adaptation loss function can be modified as

$$L_{\mathrm{multi}‐\mathrm{img}}=‐\underset{i,k,u,v}{\sum }[D_{i}\log p_{\left(i,k\right)}^{\left(u,v\right)}+(1-D_{i})\log(1-p_{\left(i,k\right)}^{\left(u,v\right)})]$$ (1)

where *D*_*ii*_ denotes the domain label of the i-th training image. *D*_*i*_ = 0 for the source domain and *D*_*i*_ = 1 for the target domain. *i* denotes the *i*-th image. *k* represents the *k*-th layer. (*u*, *v*) denotes the location in the feature maps of the domain classifier. $p_{\left(i,k\right)}^{\left(u,v\right)}$. denotes the output of the domain classifier where activated at location (*u*, *v*) of the *i*-th image in the *k*-th layers.

The multiple instance-level adaptation loss can be modified as

$$L_{\mathrm{multi}‐\mathrm{ins}}=‐\underset{i,k,j}{\sum }[D_{i}\log p_{i,k,j}+(1-D_{i})\log(1-p_{i,k,j})]$$ (2)

where *i*,*k*,*D*_*i*_ dote the same as the Eq(6). *j* means the *j*-th region proposal. *p*_*i*,*k*,*j*_ represents the output of the instance-level domain classifier for the *j*-th region prosal in the *i*-th image at *k*-th layer.

Furthermore, consistency regularization should also be modified to satisfy the multiple level adaptation. We donotes the *k*-th layer of the *i*-th image in image-level domain classifier representation as *I*_*ik*_. And we take the average over all activations in the representation as its *k*-th layer of the *i*-th image in image-level probability. The multiple consistency regularizer can be modified as:

$$L_{\mathrm{multi}‐\mathrm{cst}}=\underset{i,k,j}{\sum }\|\frac{1}{\left|I_{ik}\right|}\underset{u,v}{\sum }p_{\left(i,k\right)}^{\left(u,v\right)}-p_{i,k,j}{\|}_{2}$$ (3)

where the *|I*_*ik*_| denotes the total number of activations in the *k*-th layer of the *i*-th image, and ||·|| is the L2 distance.

Then the overl training loss is the sum of detection loss and adaptation loss, which can be modified as:

$$L=L_{\det}+\lambda (L_{\mathrm{multi}‐\mathrm{img}}+L_{\mathrm{multi}‐\mathrm{ins}}+L_{\mathrm{multi}‐\mathrm{cst}})$$ (4)

where *λ* is a trade-off parameter to balance the detection loss and adaptation loss. The detection loss *L*_det_ is the same as the original Faster R-CNN. The standard SGD algorithm can optimize the total loss in an end-to-end manner.

Faster R-CNN ] with VGG16 [28] is used as the primary detection model in our experiments. All the domain adaptation improvements are used to do adversarial training. In the testing phase, these improvements can be removed. In other words,he original Faster R-CNN architecture with adapted weights can be used to predict the bounding box and classes of objects in the testing phase. Thereupon, the testing time is not affected by our proposed improvements.

### 3.6. Loss function parameter optimized with CS

The trade-off parameter *λ* is set as 0.1 in the total loss function to balance the detection loss anadaptation loss based on the experience. However, this value is not the optimal global one. The realization of finding the best value for the trade-off parameter is a difficult problem. Usually, the grid search method is one of the solutions to find the best value of *λ*. Nevertheless, the value of the searching step is hard to define in the grid search method. The optimal results cannot be obtained when the value of the searching step is not right. In other words, the exploration ability is effected by the searching step.

**Fig 6:**
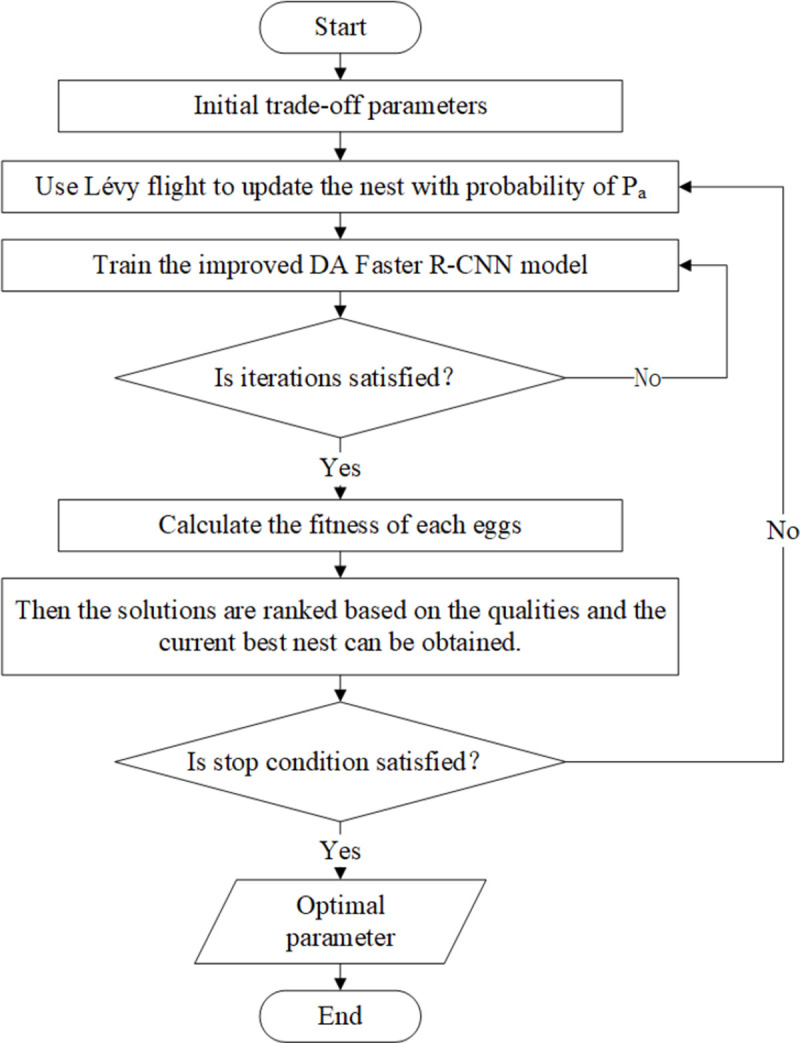
The flowchart of parameters optimization process with CS.

In this paper the optimization method CS is introduced to optimize the trade-off parameter *λ*. Cuckoo eggs represent the parameter. In other words, each egg is one possible solution of parameter *λ*. The objective function evaluates the quality of the eggs. In this work, the object detection loss function *L*_det_ is the objective function. Firstly, many nests are built, and each nest is laid with one egg by a cuckoo. Secondly, some eggs are found by the host with the probability of *P*_*a*_. Then the hosts abandon the current nests and build new nests by the Lévy flight method. Thereafter, train the improved DA Faster R-CNN model until satisfying certain iterations. After that, the quality of the eggs in the nests is calculated by the objective function. Then the solutions are ranked based on the qualities and the current best nest can be obtained. Iterate the process until the termination conditions are met. The flowchart in Fig 6. represents the parameters optimization process through CS.

## 4. Experiments and results

In order to evaluate the effectiveness of our approach, three experiments are performed: 1) Foggy Weather Adaptation. In this part, methods for detecting objects are investigated in normal weather to that in foggy weather. 2) Synthetic Data Adaptation. In this experiment, the ability of our proposed methods are tested for the synthetic data to the actual world data. 3) Cross Camera Adaptation. In this section, our novel methods are validated for the photos under different camera setups. Additionally, the visualization of feature distribution is evaluated to support our standpoint. The experiments represent that our proposed improvement can enhance the model’s overall discriminating ability.

### 4.1. Empirical setup

#### Data sets introduction

Our proposed methods are evaluated on three domain shifts in our experiments——Cityscapes to Foggy Cityscapes, SIM10K to Cityscapes, Cityscapes to KITTI. The source training data with annotation information (bounding boxes and object categories) and the target training data without annotation are provided. Details of the data sets is shown in Table 1 and in the references [15–18].

**Table 1:** Dataset information.

Dataset	No. of categories	No. of annotated images	No. of annotated objects
Cityscapes [15]	30(8)	3475	\
Foggy CityScapes [16]	30(8)	3475	\
SIM10K [17]	1	10000	58,701
KITTI [18]	3	7481	80,256

**Cityscapes [15]** is a large-scale dataset containing diverse stereo video sequences recorded in street scenes from 50 different cities. It contains 3475 high quality pixel-level annotated frames, which 2975 images are taken as the training set and 500 images are taken as the validation set. The annotations have 30 classes in detail. But only 8 representative classes are used in our experiment: person, rider, car, truck, bus, train, motorcycle, and bicycle. What`s more, pixel-level instance annotations are transformed to bounding boxes for object detection task.

**Foggy Cityscapes [16]** derives from Cityscapes. The synthetic foggy images are generated with a fog simulation pipeline for Cityscapes. So it shares the same annotations with the Cityscapes and has the same number of classes and images.

**SIM10K [17]** contains 10000 images with bounding boxes of 58701 cars captured from gaming engine Grand Theft Auto V(GTAV).

**KITTI [18]** is a part of the KITTI Vision Benchmark Suite, which aim to develop novel challenging real-world computer vision benchmarks. It is famous in auto-driving. There are only 3 annotated classes in this dataset: car, pedestrian, and cyclist, which are the most interested objects in the driving environment. The object detection benchmark consists of 7481 training images and 7518 test images, comprising a total of 80256 labeled objects.

### 4.2 Experiment details

In the experiments, SLNO-S contains the skip-layer network and improved loss function. Additionally, SLNO method includes SLNO-S and CS optimization method. The Caffe [29] framework is applied to implement our proposed method. The VGG-16 model is used as the backbone of the faster R-CNN. The convolutional parameters are initialized through the VGG-16 model which weights are pre-trained on ImageNet. In order to make certain of the experiment results stability, each experiment is repeated for 3 times and the results are averaged.

For all experiments, the results are illustrated through the mean average precisions (mAP). By default, the shorter side of all training and test images are resized to a length of 600 pixels. The hyper-parameters are set following [5]. The network is fine-tuned with a learning rate of 0.002 for 40k iterations and then the learning rate is reduced to 0.0001 for another 20k iterations. Two images from the source domain and the target domain are feed into the network every iteration. Momentum and weight decay are used in our experiments, which are set as 0.0005 and 0.9. Without specific notation, the parameters of the CS are set as in the Table 2.

**Table 2:** The parameters of the CS.

Parameter	Description	Value
N	The number of nests	10
P_a_	the probability of the host finding cuckoo eggs	0.5
α	the step factor	1
N_Max_	Max iterations	100

### 4.3. The analysis of overall model improvements

To evaluate the overall performance of our proposed method, foggy weather adaptation experiments and cross camera adaptation experiments are made. The results and analysis are as follows:

#### 4.3.1 Foggy weather adaptation

In a real scenario, weather changes frequently. The change of weather has a great impact on the data collected by the sensor. Therefore, the autonomous driving system should perform object detection effectively in different weather conditions. In this section, our proposed methods are tested from clear weather environments to foggy environments. Cityscapes and Foggy Cityscapes datasets are used as the source domain and the target domain, respectively.

As shown in Table 3, the mAP of SLNO-S method is 14.1% higher than the baseline Faster R-CNN method. SLNO-S contains the information from lower feature maps based on our multi-level feature fusion method, which enhances the feature’s comprehensive to do domain shift. In addition, both image-level and instance-level information is considered by our proposed multi-layer domain adaptation method. Thereupon, the capability of aligning the distributions from source to target domain is strengthened by the SLNO-S method. Moreover, the parameter of the loss function is optimized by CS in our SLNO method. As a result, the mAP of SLNO is 14.3% higher than the baseline Faster R-CNN model with λ = 0.17. Especially, SLNO gets the best mAP than other compared methods. The mAP of SLNO is 0.2% higher than SLNO-S, which shows that the CS method selects best parameter λ for the training phase.

**Table 3:** Detection results for SLNO-S and SLNO on the Foggy Cityscapes test set (from Cityscapes to Foggy Cityscapes). The best AP of each object category is bold-faced (%).

Approach	Backbone	mAP	person	rider	car	truck	bus	train	mcycle	bicycle
Faster R-CNN(baseline)	VGG-16	22.0	24.4	30.5	32.6	10.8	25.4	9.1	15.2	28.3
DA-Faster [9]	VGG-16	27.6	25.0	31.0	40.5	22.1	35.3	20.2	20.0	27.1
SW-Faster [10]	VGG-16	34.8	32.3	42.2	47.3	23.7	41.3	27.8	28.3	35.4
SC-DA(Type3) [10]	VGG-16	33.8	33.5	38.0	48.5	26.5	39.0	23.3	28	33.6
DT Model [13]	VGG-16	31.5	25.4	39.3	42.4	24.9	40.4	23.1	25.9	30.4
DD-MRL [23]	VGG-16	34.6	30.8	40.5	44.3	**27.2**	38.4	34.5	28.4	32.2
MTOR [20]	Resnet-50	35.1	30.6	41.4	44.0	21.9	38.6	**40.6**	28.3	35.6
SLNO-S (ours)	VGG-16	36.1	33.1	43.8	49.2	24.8	42.2	28.9	29.7	36.8
SLNO (λ = 0.17)(ours)	VGG-16	**36.3**	**33.4**	**44.1**	**49.3**	24.9	**42.3**	29.3	**30.1**	**37.1**

Additionally, we can see that the best mAP of the truck is generated on DD-MRL and the best mAP of the train is produced by MTOR with Resnet-50 backbone. However, the greatest mAP across other categories is achieved by our proposed methods. In other words, the improvements of SLNO is practical on foggy weather adaption.

#### 4.3.2 Cross camera adaptation

We are considering that the types of camera for different datasets are different. In order to further verify the effectiveness of our proposed methods. Comparative experiments are carried out through the data collected under different camera types to verify the adaptability of our proposed methods between two real datasets. In this section, Cityscapes and KITTI are used as the source domain and target domain respectively.

Because the classification standard of categories between Cityscapes and KITTI is different, then we redistribute ‘Car’ and ‘Van’ as ‘Car’, ‘Person’ and ‘Person sitting’ as ‘Person’. Additionally, ‘Tram’ is converted to ‘Train’, ‘Cyclist’ is converted to ‘Rider’ in the KITTI dataset.

As shown in Table 4, the mAPs of SLNO-S and SLNO methods are 6.9% and 7.2% higher than the baseline Faster R-CNN respectively. Additionally, the mAPs of SLNO-S and SLNO methods are also higher than the DA Model. The results indicate that the improvements of our proposed methods are effective. Particularly, we find the proposed mAPs of SLNO-S and SLNO method are 2.6% and 2.1% lower for the Faster R-CNN for car category respectively. This is because the domain shift between the source and target domain datasets in car category is very small. Additionally, the two domain datasets are both from real world with different camera configurations. Thereupon, the test results of our proposed methods are affected by the overfitting problem. However, the mAPs of our methods are the best in other categories. In other words, our methods achieve superior performance on different datasets with different camera types.

**Table 4:** Results on KITTI, using models trained on Cityscapes (from Cityscapes to KITTI) (%).

Method	Person	Rider	Car	Truck	Train	Mean AP
Faster R-CNN(baseline)	47.87	22.0	**75.2**	12.4	12.6	34.0
DA Model [9]	40.9	16.1	70.3	23.6	21.2	34.4
SLNO-S(ours)	52.9	24.2	72.6	29.2	25.5	40.9
SLNO(λ = 0.17)(ours)	**53.1**	**24.3**	73.1	**29.5**	**25.9**	**41.2**

### 4.4. Synthetic data adaptation

With the development of computational vision, synthetic data is widely used in experiments. In order to verify the effectiveness of the proposed method with the mutilple image-level improvements, the mutilple instance-level improvements and multiple consistency regularization respectively, the synthetic data adaptation datasets are carried out in our experiment to test the comparing methods. Specifically, SIM10K is used as the source domain data, which consists of 10,000 images with annotation boundaries. At the same time, Cityscapes is used as the target domain data.

The results are summarized in Table 5. Because only the cars are annotated in SIM10K, thus only the average precision of the cars on the validation set of Cityscapes are illustrated. Particularly, the mAP of our proposed SLNO-S4 is +8.4% higher than the baseline model. This proves the improvements of the proposed SLNO-S are effective. In addition, the mAP of SLNO-S1 with multiple image-level improvements is 1.9% higher than original DA Model. Specially, the mAP of SLNO-S2 with multiple instance-level improvements is 2.2% higher than original DA Model. In other words, our proposed multiple image-level adaptation and multiple instance-level adaptation improvements can decrease the domain shift on each level effectively. Moreover, 42.4 mAP is obtained by SLNO-S3 with above two improvements. This result shows the necessary of making domain shift in both level. Based on the above two improvements, the SLNO-S4 model achieves an 42.7 mAP with additional multiple consistency regularization. Furthermore, our SLNO method achieves the best mAP comparing to other methods. The reason is that the parameter of loss function is optimized by CS methods. From the results, we can see that all our improvements promote the performance of SLNO-S and SLNO.

**Table 5:** Results on Cityscapes, using models trained on SIM10K (from SIM10K to Cityscapes) (%).

Method	img	ins	CTX	L	mimg	mins	mcons	Car AP
Faster R-CNN(baseline)								34.3
DA Model [9]	✓	✓						39.4
SW-DA [10]	✓		✓	✓				40.1
SW-DA(γ = 3) [10]	✓		✓					42.3
SC-DA(Type3) [11]								43.0
SLNO-S1(ours)	✓	✓			✓			41.3
SLNO-S2(ours)	✓	✓				✓		41.6
SLNO-S3(ours)	✓	✓			✓	✓		42.4
SLNO-S4(ours)	✓	✓			✓	✓	✓	42.7
SLNO(λ = 0.15)(ours)	✓	✓			✓	✓	✓	**43.0**

### 4.5. Optimization process of parameter λ

**Fig 7:**
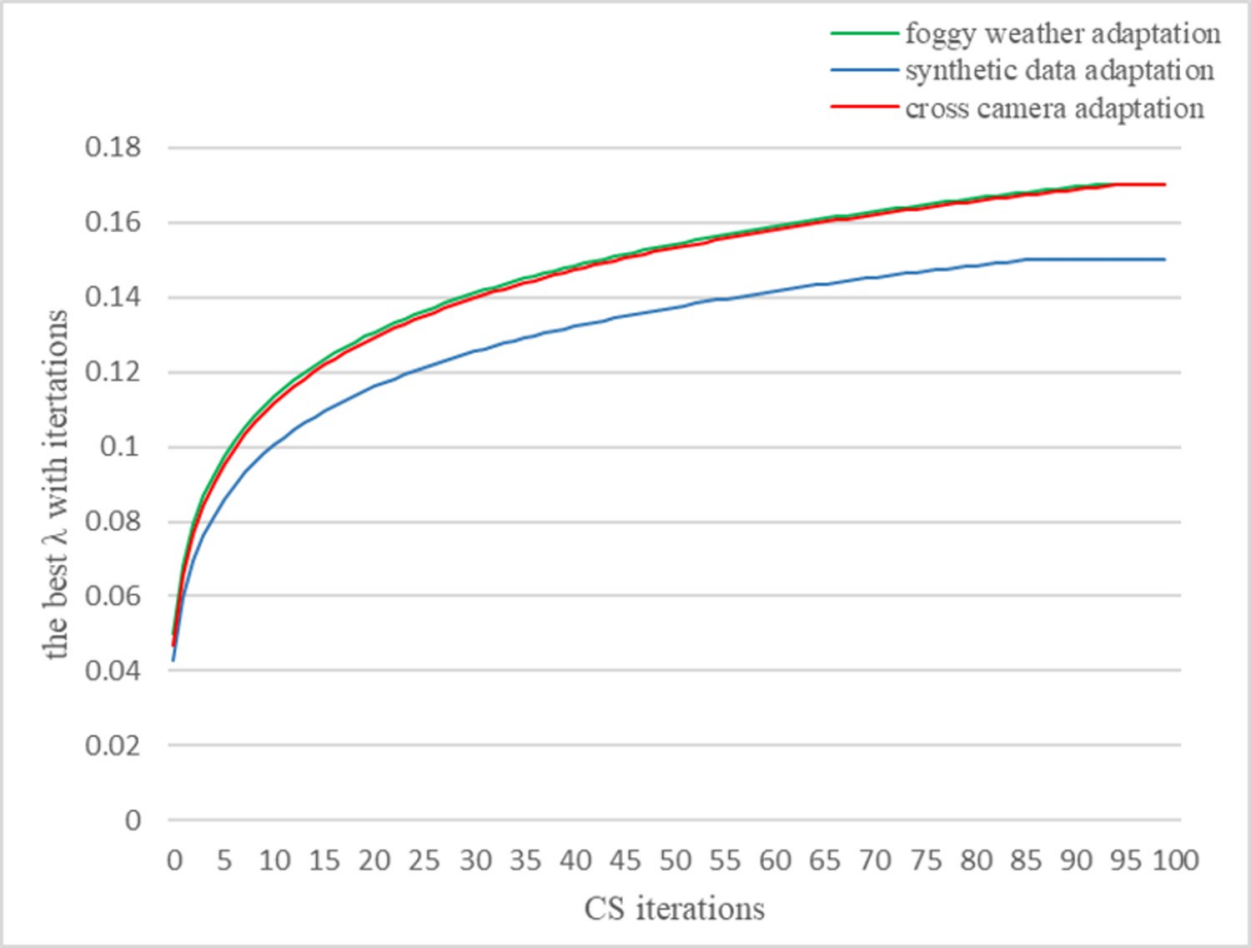
Optimization process of parameter λ.

CS method is introduced to optimize λ in the training phase. The hyperparameters for CS method are shown in Table 2. The SLNO model has trained 600 iterations and the λ is optimized once. The best λ is obtained based on increasing iterations of the CS method. The change of λ is illustrated in Fig 7.

From the Foggy Weather Adaptation, Synthetic Data Adaptation and Cross Camera Adaptation experiments, we can see that 0.17 and 0.15 are assigned to λ through CS optimization method respectively. Mainly, the parameter λ with the optimized value can promote the performance of SLNO. Therefore, the capability of SLNO is enhanced by using the optimized λ. In other words, the fixed value for λ is not the optimal value. Thereupon, the best solution for λ can be found by CS method. From Fig 7, we can see that the value of λ is small at the beginning of training. The reason is that the loss from domain adaptation should be restrained at the beginning. Furthermore, the λ converges to 0.17 when CS iterations equal to 95 in the foggy weather adaptation and cross camera adaptation experiments. Additionally, the λ converges to 0.17 when CS iterations equal to 95 in the foggy weather adaptation and cross camera adaptation experiments. However, the λ converges to 0.15 when CS iterations equal to 85 in the synthetic data adaptation experiment. The results indicate that the λ can converge to the different best values for different domain shifts.

Because synthetic data adaptation experiment has only one category, the λ in the synthetic data adaptation experiment converges earlier than that in the foggy weather adaptation and cross camera adaptation experiments. Therefore, the simple domain shift can promote the convergence of λ.

### 4.6. The analysis of modified loss function performance

We make the synthetic data adaptation experiments to evaluate the performance of modified loss function. The results and analysis are as follows:

As shown in Table 6, Lmulti-img, Lmulti-ins, Lmulti-cst denotes the multiple image-level adaptation loss function, multiple instance-level adaptation loss function, multiple consistency regularizer, respectively. Model with ✓ means the loss function is applied. In general, SLNO-S9 is the model including all modified loss function. The mAP of SLNO-S9 is +8.4% higher than the baseline model, +3.3% higher than the DA mode and +1.2% higher than SLNO-S5. This shows that the modified loss function works effectively. In details, SLNO-S5 is the model without modified loss function. The mAP of SLNO-S5 is +2.1% higher than the DA model. Because the multiple feature fusion component and multiple layer domain adaptive still works at this situation. Additionally, SLNO-S6 and SLNO-S7 is the model with the modified multiple image-level and instance-level adaptation loss function, respectively. The mAP of SLNO-S6 is 0.3% higher than the SLNO-S5, while the mAP of SLNO-S7 is 0.7% higher than the SLNO-S5. The result shows that both the modified multiple image-level and instance-level adaptation loss function are effective. And the modified multiple instance-level adaptation loss function is more effective than the modified multiple image-level adaptation loss function. Moreover, The SLNO-S8 is the model with both the modified multiple image-level and instance-level adaptation loss function at same time. The mAP of SLNO-S8 is +1.1% higher than SLNO-S5. This shows that using the modified loss function at same time could get more effective performance than using them alone. Additionally, SLNO is the model which the parameter of the loss function is optimized by CS. The mAP of SLNO get the best result at 43% with λ = 0.15. The mAP of SLNO is 0.3% higher than SLNO-S9. This shows that the CS method could selects more optimal trade-off parameter for the training phase than the set based on experience.

**Table 6:** Results with different loss function on Cityscapes, using models trained on SIM10K (%).

Method	*L*_*multi-img*_	*L*_*multi-ins*_	*L*_*multi-cst*_	Car AP
Faster R-CNN(baseline)				34.3
DA Model [9]				39.4
SLNO-S5(ours)				41.5
SLNO-S6(ours)	✓			41.8
SLNO-S7(ours)		✓		42.2
SLNO-S8(ours)	✓	✓		42.6
SLNO-S9(ours)	✓	✓	✓	42.7
SLNO(λ = 0.15)(ours)	✓	✓	✓	**43.0**

## 5. Conclusion

In this paper, our proposed Skip-Layer Network with Optimization method is introduced to domain adaptive object detection. A stable and efficient object detector can be trained by our proposed approach. The performance of the detector could be improved by align different domain distributions between source and target domain. Three improvement are proposed in this manuscript. Firstly, Multi-level feature fusion is designed to enhance the features from lower-level feature maps. Fused features are benefit for classification. Secondly, multi-layer domain adaptation is proposed to align the domain shift in image-level and instance-level by skip layer. The loss functions are modified to fit the multiple structures. Thirdly, CS method is applied to optimize trade-off parameters in the training phase and makes the performance better. Our approach is validated on three domain shift scenarios, which named foggy weather adaptation, cross camera adaptation and synthetic data adaptation. The results of all experiments show that our proposed methods outperform baseline Faster R-CNN and DA models significantly. the In other words, the improvements in SLNO could promote the performance of cross-domain object detection.
